# Clinical implementation of a one-step no-wash flow cytometry method allows for real-time monitoring of patients treated with autologous CAR-T cells

**DOI:** 10.3389/fonc.2026.1774431

**Published:** 2026-04-10

**Authors:** Nassim Salem, Clémence Demerlé, Bechara Mfarrej, Ludovic Blaison, Pierre Lignée, Jérôme Couquiaud, Sarah Ouffai, Carine Malenfant, Thibaut Reichert, Julie Demaret, Jacques Trauet, Ibrahim Yakoub-Agha, Faezeh Legrand Izadifar, Angela Granata, Sophie Thévenet, Anne-Line Château, Boris Calmels, Christian Chabannon, Gabriel Brisou, Claude Lemarié

**Affiliations:** 1 Centre de Thérapie Cellulaire, Département de Biologie du Cancer, Institut Paoli-Calmettes, Marseille, France; 2 Module Biothérapies du Centre d’Investigation Clinique de Marseille, AP-HM, Aix-Marseille Université, Institut Paoli-Calmettes, CBT-1409, INSERM, Marseille, France; 3 Département de Médecine Nucléaire, Institut Paoli-Calmettes, Marseille, France; 4 Institut d’Immunologie, CHU Lille, Lille, France; 5 U1286-Infinite-Institute for Translational Research in Inflammation, Université de Lille, INSERM, CHU Lille, Lille, France; 6 Unité d’Allogreffe, Maladies du sang, CHU Lille, Lille, France; 7 Département d’hématologie, Institut Paoli-Calmettes, Marseille, France; 8 Faculté des Sciences Médicales et Paramédicales, Aix-Marseille Université, Marseille, France

**Keywords:** CAR-T cells, cellular therapies, immune monitoring, immunotherapy, lymphoma, method validation

## Abstract

**Background:**

The best way to monitor Chimeric Antigen Receptor (CAR)-T cells persistence and expansion *in vivo* after infusion, and the significance of observed fluctuations over time remains controversial. As living drugs, these therapies do not follow classical pharmacokinetic patterns. Therefore, reliable quantification of circulating CAR-T cells is essential to explore the relations between *in vivo* expansion and persistence on one hand, tumor response and occurrence of side-effects on the other hand; this work will prepare integration of this information in harmonized post-CAR-T Cells intervention algorithms. Conventional flow cytometry protocols rely on multi-step wash procedures that increase processing time and may reduce sensitivity. We here developed and validated a one-step no-wash flow cytometry assay for routine CAR-T monitoring in patients treated with approved autologous CAR-T Cells.

**Methods:**

CAR-T cell monitoring was implemented between 2021 and 2024 in patients treated with autologous CD19-directed CAR-T therapies at our institution with a classical two steps and wash flow cytometry method. Analytical validation included determination of detection and quantification limits, linearity, precision, and inter-laboratory reproducibility. In 2024, the classical method was considerably optimized to a one-step no-wash format to reduce manual handling and improve sensitivity. Clinical relevance was assessed in a cohort of 29 patients treated with axicabtagene ciloleucel, correlating CAR-T expansion metrics with clinical endpoints.

**Results:**

The optimized one-step no-wash assay markedly improved analytical sensitivity, achieving a limit of detection of 0.3 cells/µL and a lower limit of quantification of 1.0 cells/µL, versus 2.0 and 5.0 cells/µL, respectively, when using the previous two steps and wash protocol, while demonstrating a strong concordance (r² = 0.984). Inter-assay coefficients of variation remained below 9%, confirming maintained precision despite workflow simplification. In 29 patients treated with axicabtagene ciloleucel (axicel), peak CAR-T expansion significantly correlated with objective responses (p< 0.01) and occurrences of immune effector cell–associated neurotoxicity syndrome (ICANS) (p = 0.02), supporting the clinical relevance of flow cytometry–based CAR-T monitoring in routine practice.

**Conclusion:**

This one-step no-wash flow cytometry assay combines enhanced sensitivity with improved operational efficiency and provides clinically informative CAR-T cell monitoring in standard-of-care settings.

## Background

CAR-T cells are emerging as a standard of care for several relapsed/refractory (r/r) B-cell lymphoid disorders including Diffuse Large B-Cell Lymphoma (DLBCL), Acute Lymphoblastic Leukemia (ALL) and Multiple Myeloma (MM). Although Axicabtagene ciloleucel (axicel) and Tisagenlecleucel (tisacel) were initially approved for third-line patients, the recent findings from the ZUMA-7 and TRANSFORM randomized phase 3 trials have demonstrated superior outcomes for patients treated with axicel and lisocabtagene maraleucel (liso-cel), respectively, compared to salvage chemotherapy and high-dose consolidation therapy with autologous hematopoietic cell transplantation (1, 2). This development opened the door for the use of autologous CAR-T cells as a second-line option for r/r DLBCL. More recently anti-B-Cell Maturation Antigen (BCMA) CAR-T cells such as idecabtagene vicleucel (idecel) and ciltacabtagene autoleucel (ciltacel) were approved in third line treatment for patients suffering from multiple myeloma that is resistant to the three major classes medicinal products used to treat plasma cell disorders.

Nonetheless, not all patients benefit from this treatment, and the early identification of responders vs non-responders would greatly improve patients care. A second critical point is the prediction of CAR-T specific toxicities such as Cytokine Release Syndrome (CRS) or Immune effector Cell-Associated Neurotoxicity Syndrome (ICANS), helping in deciding on early therapeutic interventions. Therefore, biomarkers may help for refining patient selection and adjusting treatment at an early stage to enhance the long-term effectiveness of this therapy. Numerous clinical and biological parameters have been discussed but post-infusion circulating CAR-T cells concentrations remain a key biological parameter directly reflecting patient exposition to the therapy.

CAR-T cell expansion in peripheral blood is monitored in most clinical trials as a secondary endpoint using flow cytometry or RT-qPCR (3). A higher CAR-T cell expansion is associated with an improved response rate to axicel (4–11). However, multiple factors may affect the precision of these monitoring; one such factor includes the need for these methods to be standardized and approved so that the results can be used in clinical practice (12).

Also, Locke et al. (8, 9) and others have established a statistical correlation between the clinical response of CAR-T treatment and the composition of infused CAR-T cell products. As described for hematopoietic cellular products (13), a single-platform method using a viability dye could increase robustness and precision cellular quantification. Our team has a recognized expertise in technical validation of single-platform flow cytometry based assays, and we previously reported on several method validations to monitor cells by cytometry or evaluate cellular product potency (14–16).

We here report on the biological validation of single-platform flow cytometry techniques to monitor viable CAR-T cells concentrations in the peripheral blood of patients, based on International Organization for Standardization (ISO) 15189 criteria (17). In line with regulatory standards requirements, we evaluated the precision, intermediate fidelity, Limit of Detection (LOD) and Quantification (LLOQ), and performed an inter-laboratory comparison in absence of commercialized quality control. The technique established in 2020 allowed us to compare biological findings and several clinical endpoints for the first 29 patients treated by axicel at our hospital between Jun 2021 and July 2023. We also share the updated methodology recently implemented with a new cytometer allowing a single-step staining and an extended panel.

With this standardized and easily reproducible protocol, we demonstrate that CAR-T cells monitoring could be part of routine laboratory medicine in real-world clinical practice and fully integrated in patient’s follow-up after CAR-T cell infusion. In the longer term, better-defined endpoints to inform clinical decisions will emerge from the growing body of clinico-biological data.

## Methods

### Sample management

Fresh blood samples from CAR-T cell treated patients were collected in Ethylenediaminetetraacetic acid (EDTA) tubes. All informed consent forms are available on records. Samples were diluted, if necessary, in Phosphate-buffered saline (PBS) to a final concentration not exceeding, 20 x10^6^ white blood cells/mL.

### First method (2020): two-step methodology

100 µL of blood sample were stained with 5µL of CD19 CAR-T detection reagent (130-129–550 Miltenyi Biotec, Bergisch Gladbach, Germany) during 10 minutes at Room Temperature (RT) in the dark. Sample was then washed with 1ml PBS and centrifuged 5 minutes at RT. The cell pellet resuspended in 50 µL was then transferred in TruCount tubes (BD) and stained with the second mix containing the Streptavidin Conjugate PE-Cy7 (130-106-793) to reveal CAR-T primary staining, the following gating antibodies: CD3-PE (clone UCHT1, A07747), CD45-FITC (clone J33, A07782) and 7-Aminoactinomycin D (7-AAD) viability dye (A07704) during 20 minutes at RT in the dark, all reagents coming from Beckman Coulter (Beckman Coulter, Marseille, France). All lot numbers are retained on records. For sample erythrolysis, 450 µL of 1X IOTest3 lysing solution (PN IM3514) was added to each tube, and incubated during 10 minutes in the dark at RT. Sample acquisition was performed immediately on a Canto II cytometer (BD Biosciences), data were analyzed with Diva Software (BD Biosciences), The beads in the Trucount (i.e. Beads per pellet, BD) are used to calculate the concentration in absolute value for every population of interest. Anti-CD19 CAR-T cells were defined as immune (CD45+) live (7AAD-) T cells (CD3+) with positive staining for CAR (CAR+). The acquisition was stopped when 10,000 CD3+ cell events were recorded or after 6 minutes.

All Samples were stained and analyzed within 12 hours of sample collection. Samples from patients with less than 0.5x10^6^ WBC/mL were not processed.

### Updated method (since 2024): single-step methodology

This method was set to provide faster and simpler techniques to face the growing number of CAR-T cells monitoring prescription, and to monitor easily both BCMA and CD19 CAR-T cells.

We prepared ahead of time a combination of the following antibodies from BD Biosciences: CD3 APC (clone SK7, 345767), CD45 V500 (clone HI30, 560777), CD4 V450 (clone SK3, 651849), CD8 APCH7 (clone SK1, 560179) and a viability reagent 7AAD (559925) which is common for both CD19 and BCMA CAR-T monitoring. 20 µL of this ready-to-use mix was added to 50 µL of blood sample directly in the Trucount Tube (BD). New CAR detection reagent (CDR) directly coupled to fluorochrome, either BCMA (BCMA CDR-PE, Miltenyi Biotec 130-133-888) or CD19 (CD19 CDR α-FMC63-PE, Miltenyi Biotec 130-127-342) was added extemporaneously in the analytical tube. After 20 minutes of staining at RT in the dark, no washing step was necessary and gentle sample erythrolysis was performed using 450 µL of an ammonium-chloride based lysing solution (PharmLyse, BD Biosciences, 555899), All antibodies were previously titrated to optimize the staining and reduce the cost. All antibodies contained 0.1% sodium azide. All lot numbers are retained on records. Stability of the mix was established for at least 1-month duration.

Sample acquisition was performed on a 10 color-Lyric cytometer (BD Biosciences), data were analyzed with BD FACS Suite Software (BD Biosciences). CAR-T cells were defined as CD45+/7AAD-/CD3+/CAR+ cells (BCMA or CD19 respectively) and further delineated in the two subpopulations of CD4+ CAR-T cells or CD8+ CAR-T cells.

### WBC control

For each sample analyzed with either protocol, we verified the accuracy of flow cytometry absolute counts using an automated hematology analyzer (XP-300, Sysmex). The total CD45+ cell concentration measured by flow cytometry was compared with the white blood cell (WBC) count obtained on the hematology counter. A discrepancy of less than 20% between the two measurements was required. This quality control step ensured that manual procedures, particularly pipetting, dilution, and washing, were performed correctly since these steps are critical for reliable absolute quantification.

### Method validation

The ISO 15189 standard requires the evaluation of several performance criteria to ensure the reliability of a measurement method. These criteria include intra-assay and inter-assay precision, limits of quantification, and inter-laboratory comparisons.

Intra-assay precision is assessed under conditions that involve the same measurement procedure, the same operators, the same instrument, identical operating conditions, and the same location, with repeated measurements performed on the same sample within a short period. This parameter reflects the intrinsic variability of the measuring system. In our study, intra-assay precision was evaluated using five samples, each processed in triplicate by a single technologist on the same day. Stabilized blood samples are not available for CAR-T cells, so we relied on real samples from peripheral blood and thawed CAR-T cell products. For this reason, all three replicates for each sample had to be completed within a few hours, as recommended by Wood and colleagues (18).

Inter-assay precision is assessed using the same measurement procedure and location but across an extended time frame, with potential variation in operators. This analysis reflects the variability of the method itself. Using fresh samples, inter-assay precision was determined with five to seven samples for the two protocols, each processed in triplicate by three different technologists on the same day, following the approach described by Wood et al. (18).

The limit of quantification represents the lowest concentration that can be measured with acceptable reliability and defined uncertainty. We established this limit using 30 negative samples for the two-step method and 10 negative samples for the single-step method, which allowed estimation of background noise and determination of the lower limits.

Method comparison was performed through an inter-laboratory assessment with the Laboratory Medicine Department at Lille University Hospital. We compared both of our methods with their validated assay based on FITC-labelled CD19 and BCMA human proteins (His Tag, CD9-HF2H2 and BCA-HF2H1, Acro Biosystems, Newark) (19).

### Patient management

The clinical study was approved by the institutional review board (GSPC) at Institut Paoli-Calmettes (IPC) Comprehensive Cancer Centre and was conducted in accordance with Good Clinical Practice guidelines of the International Conference on Harmonization (ICH). All studies were performed according to institutional and Helsinki guidelines regarding human ethics. All included Patients were treated in the Department of Oncohematology at IPC, which is a qualified center for commercialized CAR-T cells administration, using institutional guidelines and Standard Operating Procedures (SOPs).

All patients had histologically confirmed large B-cell lymphoma, mostly including DLBCL, based on the 2016 World Health Organization guidelines. Among them, we focused our study on patients treated in 2^nd^ and 3^rd^ line of treatment with YESCARTA™ (axicabtagene ciloleucel, Axicel, Kite, Gilead, Santa Monica, California) between September 2021 and June 2023. Starting 5 days before CAR T-cell infusion, all patients received a standard lymphodepletion regimen (fludarabine 30 mg/m²/day and cyclophosphamide 500 mg/m²/day for 3 days). One hour prior infusion, they also all received a premedication of paracetamol 1g and Dexchlorpheniramine IV 1g as it is preconized in best practice recommendations of the European Society for Blood and Marrow Transplantation (EBMT) and the Joint Accreditation Committee of ISCT and EBMT (JACIE) (20).

Circulating CAR-T cells monitoring after infusion was performed at D3, D7, D10, D15, D21 and D28 pre-established with clinicians with our validated technique (two-step) at the time.

Patients were classified as “Responders” or “Non-Responders”, based on positron emission tomography (PET) analyses 3 months after CAR-T cells infusion. Fluorodeoxyglucose–PET (FDG-PET) imaging using the 5-point Deauville score is the gold-standard assessment for determining the end-of-treatment response in DLBCL (21, 22). Early imaging evaluation correlates strongly with clinical endpoints (Progression-Free Survival and Overall Survival) and is used in clinical practice to guide clinicians’ decision on retreatment (23–25). Consistently with the literature on post CAR-T cells follow-up, we classified “Responders” as Deauville 1 to 4 patients, and “Non-Responders”, with Deauville 5 for the Objective Response Rate (ORR) assessment (26). CAR-T cells toxicities such as Cytokine Release Syndrome (CRS) and Immune effector cell-associated neurotoxicity syndrome (ICANS) were assessed for each patient and graded from I to IV according to severity of each syndrome.

### Statistical analyses

Data were expressed as median and inter-quartile range (IQR). A two-tailed Mann-Whitney test was used when comparing two groups. For inter-laboratory comparisons, Bland-Altman graphs were used to compare frequencies of CAR-T cells within CD3+ T cells. Absolute CAR-T cell counts could not be compared due to technical heterogeneity between the two laboratories (Dual-platform in Lille versus single-platform in Marseille).

Coefficient of variation (CV) is reported as percentage of standard deviation (SD) to the mean. Statistical analyses were done using GraphPad Prism 8, version 8.02 (GraphPad). p-values ≤ 0.05 were considered significant.

## Results

### First method

#### Gating strategy

**Figure 1:**
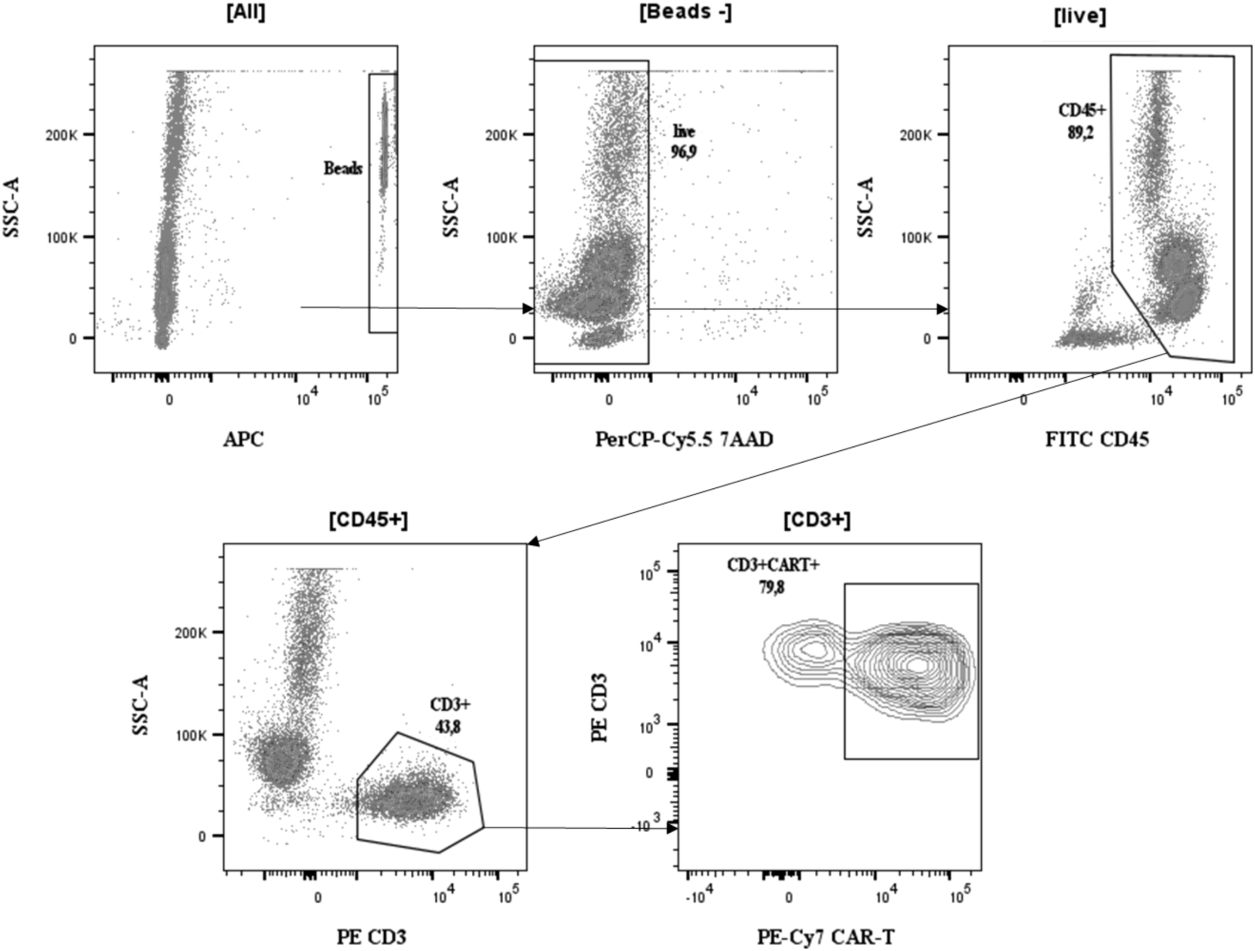
Gating strategy for the two-step method. Absolute counting beads were first identified and excluded based on forward scatter, side scatter, and fluorescence characteristics. Dead cells were excluded using 7-aminoactinomycin D (7-AAD) staining. CD45-positive leukocytes were then selected. Within this population, CD3-positive T cells were gated, and CAR-positive events were identified using the CAR detection reagent. Absolute quantification was calculated using TruCount beads according to the manufacturer’s formula.

Previously published French and German reports (14, 17–21) compared several techniques in terms of sensitivity and specificity. We developed a flow cytometry staining procedure to detect anti-CD19 CAR-T cells in the blood of patients after infusion as well as in the thawed CAR-T cell product, using the Miltenyi’s two-step procedure (27). After beads counting and exclusion (based on size and APC-fluorescence signal), dead cells were excluded with 7-AAD staining. As previously described (28, 29), leucocytes were gated based on CD45 expression (**Figure 1**). Within the leucocyte gate, lymphocytes were identified as high CD45 expression and low granularity (SSC). T-lymphocytes were identified as CD3+/CD45+ cells. CAR-T cells were identified as T lymphocytes expressing a CD19 or BCMA-binding protein. As an internal negative control, we used CD45+/CD3- cells as they cannot express a CAR due to the manufacturer’s transduction method.

Absolute quantification was done using the following formula:


$$\begin{array}{l} Absolute\;number\;of\;CAR-T\;cells\;\left(\frac{cells}{µL}\right)\\ =\frac{\#\text{events }\left[\text{CAR T gate}\right]\;\times \;\#\text{Beads in the TruCount tube }\times \text{ Dilution factor}}{\#\text{events }\left[\text{Beads gate}\right]\;\times \text{ volume}} \end{array}$$

As mentioned previously, we systematically performed a leucocyte counts with our hematology counter (XP-300 Sysmex) as a control for the CD45+ cell count measured by flow cytometer.

#### Precision

Precision describes the closeness of individual measures of an analyte, when the assay is repeatedly applied, in a single batch by the same operator (intra-assay) or across several batches by different operators (inter-assay). Intra- and inter-assay precision were calculated as recommended by Wood & al (18).

Intra-assay precision was evaluated using 5 different samples (2 thawed CAR-T cell products and 3 blood samples), handled in triplicate by the same lab technologist over a few hours to avoid the possibility of time affecting cell viability. The acceptance criterion was set at 10% as described by Wood & al (18).

Inter-assay precision was evaluated using 4 different samples (1 thawed CAR-T cell product and 3 blood samples), handled in triplicate by three different lab technologists over a few hours. The acceptance criterion was set at 13.3% as it corresponds to 1.33 times the CV of the intra-assay precision (30).

With this two-step technique CV percentages in both absolute quantification (CAR-T cells/µL) and percentages within T lymphocytes (%CD3+) fall within the predefined acceptance criterion of<10% and<13.3%, respectively, for intra (**Table 1**) and inter-assay precision evaluation (**Table 2**).

**Table 1:** Intra-assay precision of the two-step method.

Sample	CAR-T cells (cells/µL)		% CAR-T cells (within CD3+)	
	Mean ± SD	CV %	Mean ± SD	CV %
CAR-T cell product*	1685 ± 111.0	6.6%	26.7 ± 0.9	3.2%
	200 ± 6.7	3.4%	15.4 ± 0.5	3.0%
Blood	37± 2.9	7.9%	40.3 ± 0.6	1.4%
	211 ± 1.4	0.7%	22.2 ± 0.4	1.8%
	200 ± 9.0	4.5%	24.5 ± 1.2	4.7%

Five samples (two thawed CAR-T products and three blood samples) were processed in triplicate by the same technologist. Results are expressed as mean ± standard deviation (SD) with coefficient of variation (CV). Acceptance criterion was CV< 10%. The CV and SD of the replicates were calculated for absolute count and frequencies of CAR-T cells within CD3 +.

*CAR-T cell product consists in the residual cell suspension obtained through flushing of the thawed bag and tubing before they are discarded, after axicel infusion to the patient.

**Table 2:** Inter-assay precision of the two-step method.

Method	Sample	CAR-T cells (cells/µL)		% CAR-T cells (within CD3+)	
		Mean ± SD	CV %	Mean ± SD	CV %
Two-step	Blood	29.4 ± 0.4	1.4%	8.4 ± 0.2	2.4%
		27.3 ± 0.6	2.1%	3.9 ± 0.4	10.3%
		9.5 ± 0.5	5.3%	16.8 ± 1.0	6.4%
	CAR-T cell product	21.1 ± 2.7	12.8%	6.1 ± 0.6	10.5%

Four samples were processed in triplicate by three different technologists. Results are expressed as mean ± SD with CV. Acceptance criterion was CV< 13.3%. The CV and SD of the replicates were calculated for absolute count and frequencies of CAR-T cells within CD3 +.

To sum up, these data show an acceptable assay precision, supporting confidence in reporting meaningful CAR-T cells quantification and accounting for negligible inter-operator variations.

### Sensitivity and lower limit of quantitation

**Figure 2:**
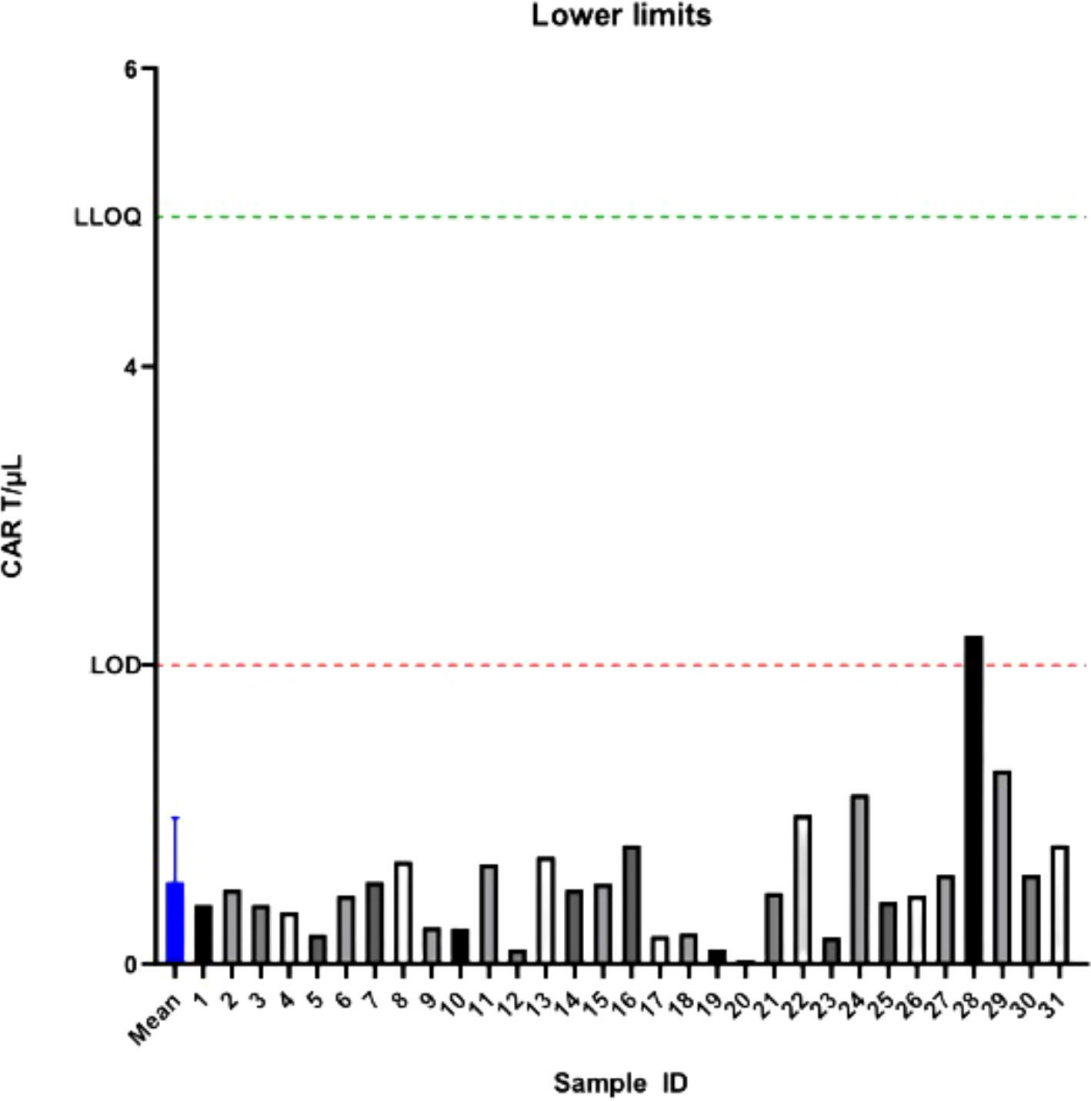
Determination of the limit of detection (LOD) and lower limit of quantification (LLOQ) for the two-step method. CAR-T cell absolute counts were measured in 31 negative control samples from patients not treated with CD19 CAR-T cells. The mean background signal is represented by the blue line. LOD was defined as mean + 3 standard deviations (SD), and LLOQ as mean + 10 SD.

In cases where the number of circulating CAR-T cells is exceedingly low, both in samples taken shortly after infusion and long afterwards, it is important to establish the lower limit of quantification. This lower limit should be defined so that its total error aligns with our predetermined acceptance criterion. To determine the Lower Limit of Detection (LOD) and Lower Limit of Quantification (LLOQ) of anti-CD19 CAR-T cell monitoring technique, we measured the absolute number of CAR-T cells in the blood of 31 patients not treated by CD19 CAR-T cells (**Figure 2**). Using this method, we set up both the Lower Limit of Quantification (LLOQ), which corresponds to Mean of the blank + 10 SD and the Limit of Detection (LOD), which corresponds to mean of the blank + 3 SD. We identified 2.0 cells/µL and 5.0 cells/µL as LOD and LLOQ respectively for the two-step method (**Figure 2**).

### Inter-laboratories comparison

**Figure 3:**
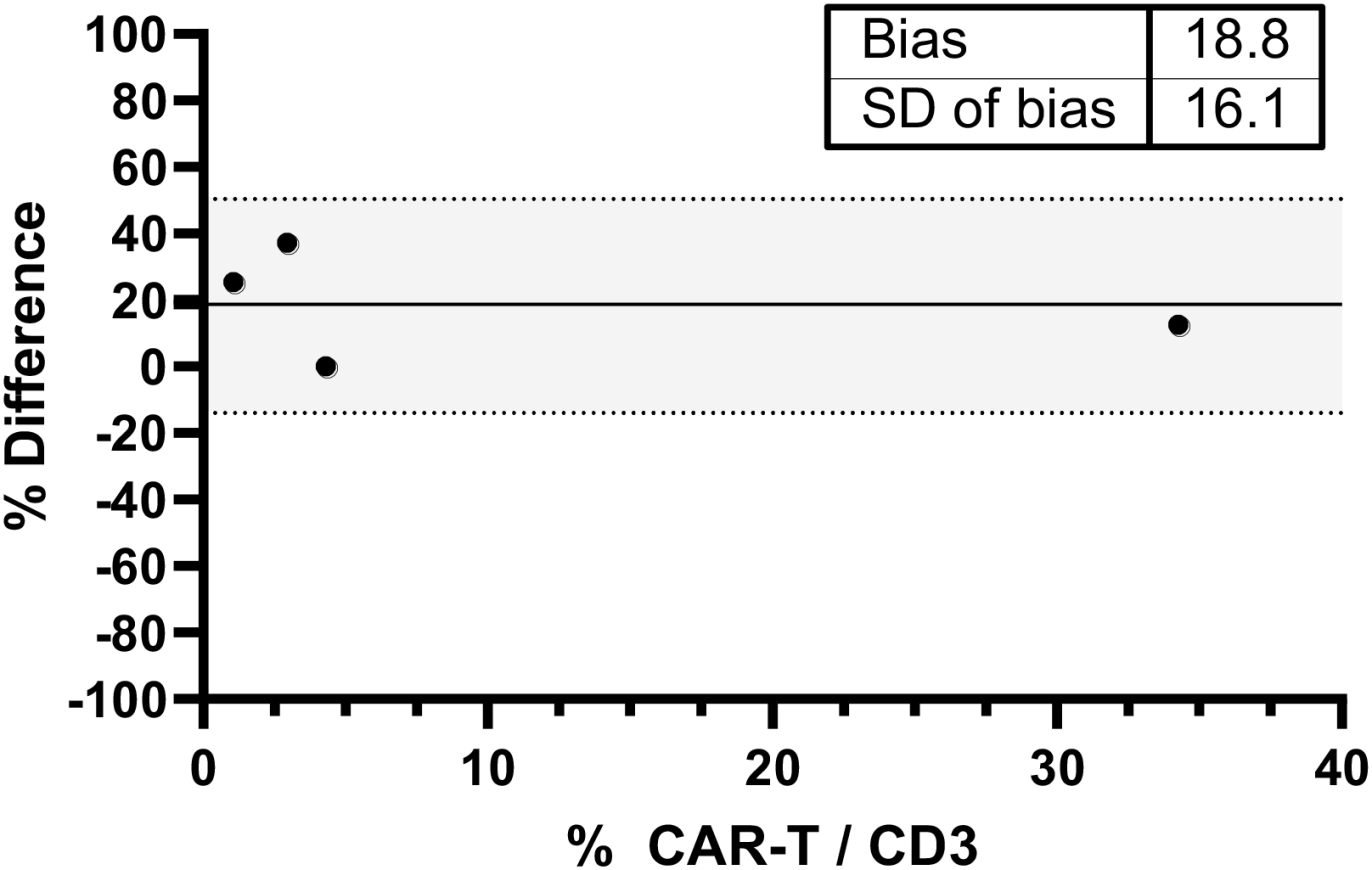
Inter-laboratory comparison of CAR-T cell frequencies between Marseille and Lille laboratories. Bland–Altman analysis was performed on six paired samples. The x-axis represents the mean CAR-T cell frequency (% within CD3+ T cells) obtained by both laboratories, and the y-axis represents the difference between measurements. The solid line indicates the mean difference, and dashed lines represent the limits of agreement defined as mean ± 2 standard deviations. All values fell within predefined acceptable limits.

An external validation is needed as required by the International Standard ISO 15189. As External Quality Assessments (EQA) are currently unavailable for CAR-T cell monitoring, we set up an inter-laboratory comparison protocol (31). Therefore, we exchanged samples with the Immunology Laboratory at Lille University Hospital (**Figure 3**). Since the two absolute counting methods significantly differ (single vs dual platform respectively), we only compared CAR-T cells frequencies. All results fell within acceptable values identified (± 2 SD).

In the absence of commercialized EQA, theses Inter-Laboratories Comparisons were the only alternatives to validate the accuracy of our techniques. Discussions are in progressed to federate a national consortium about performing these controls at a larger scale.

### Patients

Thirty-five patients treated with axicel were included in this study. Patient characteristics are described in **Table 3**. The maximum concentration (Cmax) was defined as the highest CAR-T cell number measured between Day 6 and Day 15 after CAR-T cell infusion. The Cmax was available for 28 patients (80%). The CAR-T cell peak was either under the LOD (for 2 patients, 6%) or unmeasured during the target period (6 patients, 17%). Among them, 18 are “Responders” (64%) and 10 “Non-Responders” (36%). Median follow-up was 323 days (23-933).

**Table 3:** Patient characteristics and clinical outcomes.

Patients included	35
Age (range)	62.2 (37-79)
Sexe Ratio (H/F)	23/12
CAR-T cell biological monitoring	
Number of CAR-T dosages per patient (range)	3 (1-9)
C max availability	28
Clinical responses	
Responders	18
Non responders	10
Toxicities	
CRS (Gr I-II/Gr III-IV)	16/12
ICANS (Gr I-II/Gr III-IV)	24/4
Median Follow-up in days (range)	323 (23 – 933)

Demographic data, number of CAR-T cell measurements, clinical responses, toxicities, and follow-up duration are summarized.

### Patient Demographics and clinical characteristics

### Axicel exposition associates with response and toxicity

**Figure 4:**
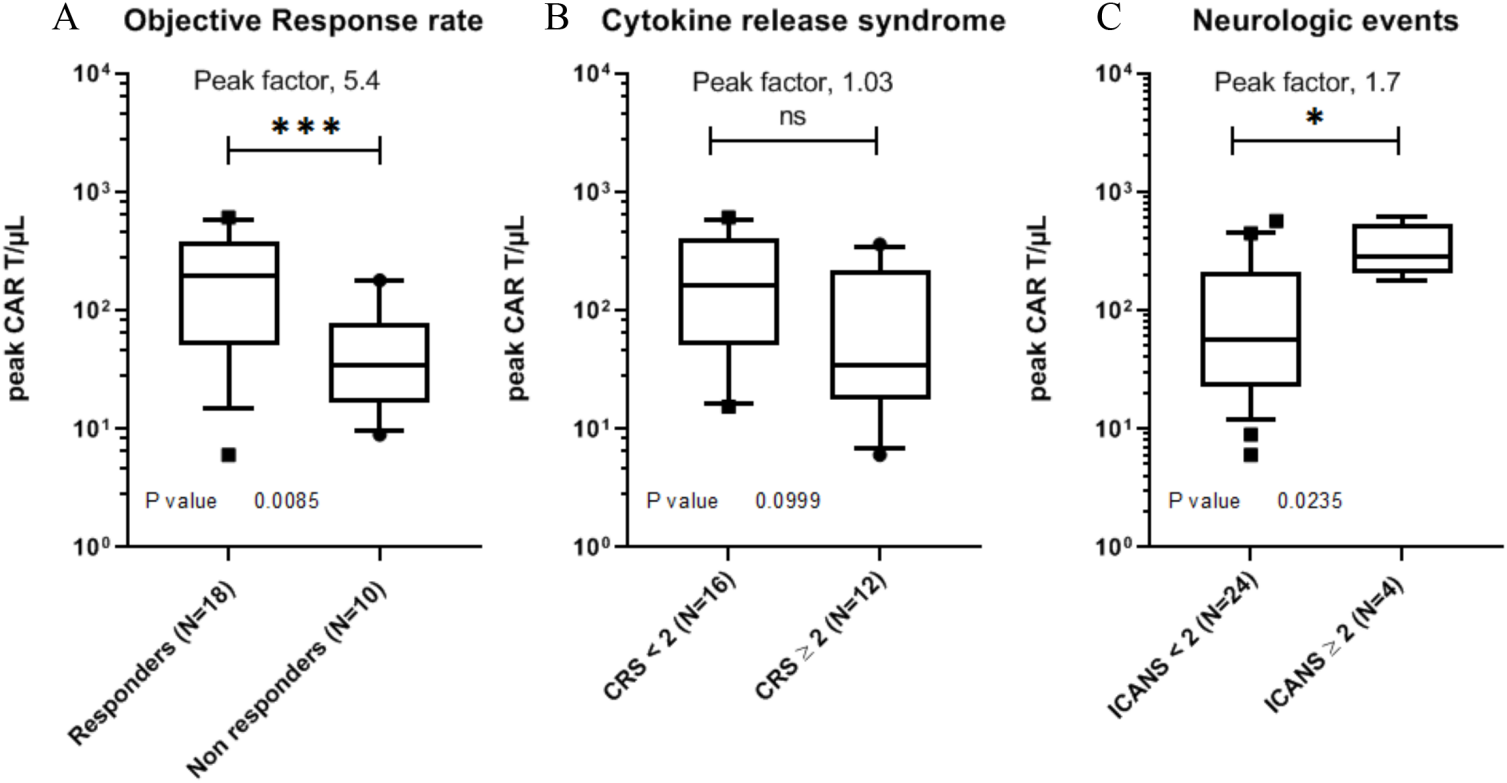
Association between peak circulating CAR-T cell concentration **(Cmax)** and clinical outcomes. Cmax was defined as the highest absolute CAR-T cell count (cells/µL) measured between Day 6 and Day 15 after infusion. **(A)** Comparison between responders and non-responders according to objective response rate. **(B)** Comparison according to cytokine release syndrome (CRS) grade ≥ 2. **(C)** Comparison according to immune effector cell-associated neurotoxicity syndrome (ICANS) grade ≥ 2. Data are presented as median with interquartile range. Statistical comparisons were performed using a two-tailed Mann–Whitney test. * p ≤ 0.05, *** p ≤ 0.001.

As in most previously published studies (4, 32, 33), CAR-T levels peaked in the peripheral blood between 7 and 10 days after axicel infusion. The Cmax was significantly associated with the objective response rates (p=0.008) and Immune effector cell-associated neurotoxicity syndrome (ICANS) of grade 2 or higher (p=0.02). Peak expansion was not associated with Cytokine release syndrome (CRS) of grade 2 or higher (**Figure 4**).

### Updated technique (2024)

With the growing interest of our clinicians for CAR-T cells monitoring we further improved our technique to monitor CD19 CAR-T cells and the recently commercialized new class of anti-BCMA CAR-T cells. In this expanded technique we have implemented directly coupled reagents to detect BCMA and CD19 CAR-T cells, and added new T markers to our panel, to reduce manual step and refine CAR-T cells characterization. This optimization was also made possible by the concomitant replacement of our former flow cytometer (BD FACS Canto II, 2 lasers) with a next-generation3-laser flow cytometer (BD FACS LYRIC).

### Gating strategy

**Figure 5:**
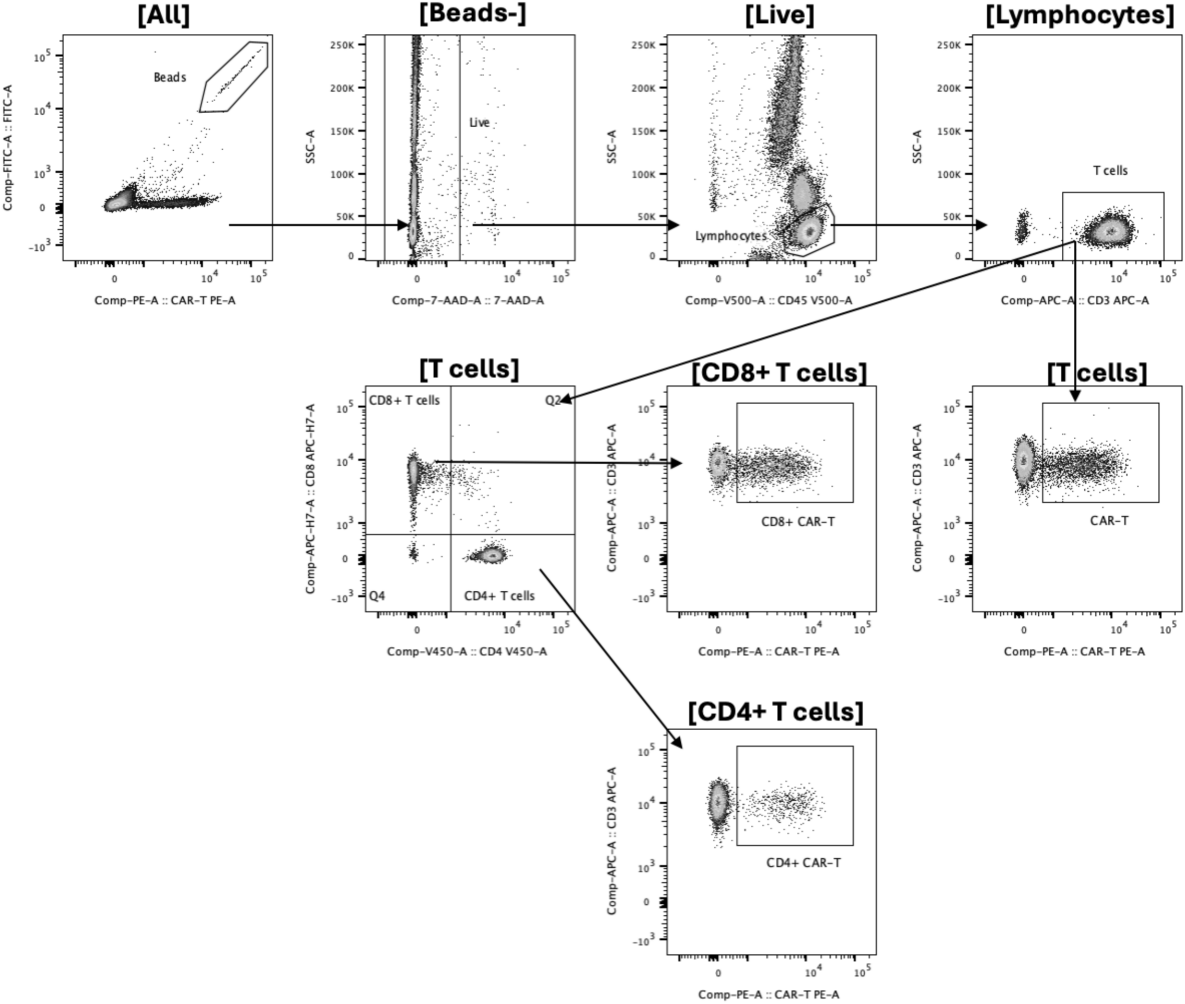
Gating strategy for the single-step method. Absolute counting beads were excluded based on scatter and fluorescence properties. Dead cells were excluded using 7-aminoactinomycin D (7-AAD). CD45-positive leukocytes were selected, and lymphocytes were identified according to side scatter (SSC) characteristics. CD3-positive T cells were gated, and CAR-T cells were defined as viable CD45+/CD3+/CAR+ events using directly fluorochrome-conjugated CAR detection reagents (CD19 or BCMA). CD4 and CD8 subpopulations were subsequently identified within the CAR-positive T-cell compartment. Absolute quantification was calculated using TruCount beads according to the manufacturer’s formula.

Our gating strategy remains similar to the first protocol. CAR-T cells were defined as living CD45 positive CD3 positive cells recognizing BCMA or CD19 CDR coupled to PE, (**Figure 5**) and subset in CD4 CAR-T and CD8 CAR-T population. We used the same absolute count method with Trucount Tubes as in the first method (See Paragraph First Method, sub-paragraph Gating Strategy),

### Inter-assay precision, one-step method

As with our two-step technique (**Table 2**), CV percentages in both absolute quantification (CAR-T cells/µL) and percentages within T lymphocytes (%CD3+) fall within the predefined acceptance criterion of<13.3%, for inter-assay precision evaluation (**Table 4**). We assumed that if the inter-assay precision for the single-step technique was as good as for the two-step protocol, evaluating the intra-assay precision was not required.

**Table 4:** Inter-assay precision of the single-step method.

Method	Sample	CAR-T cells (cells/µL)		% CAR-T cells (within CD3+)	
		Mean ± SD	CV %	Mean ± SD	CV %
Single-step	Blood	107.2 ± 9.5	8.9%	76.3 ± 2.9	3.8%
		45.9 ± 2.9	6.3%	31.0 ± 2.7	8.6%
		20.7 ± 1.6	7.7%	41.8 ± 2.0	4.7%
		5.9 ± 0.3	4.2%	39.5 ± 1.7	4.4%

Four blood samples were processed in triplicate by three different technologists. Results are expressed as mean ± SD with CV. Acceptance criterion was CV< 13.3%. The CV and SD of the replicates were calculated for absolute count and frequencies of CAR-T cells within CD3 +.

### Sensitivity and lower limit of quantitation

**Figure 6:**
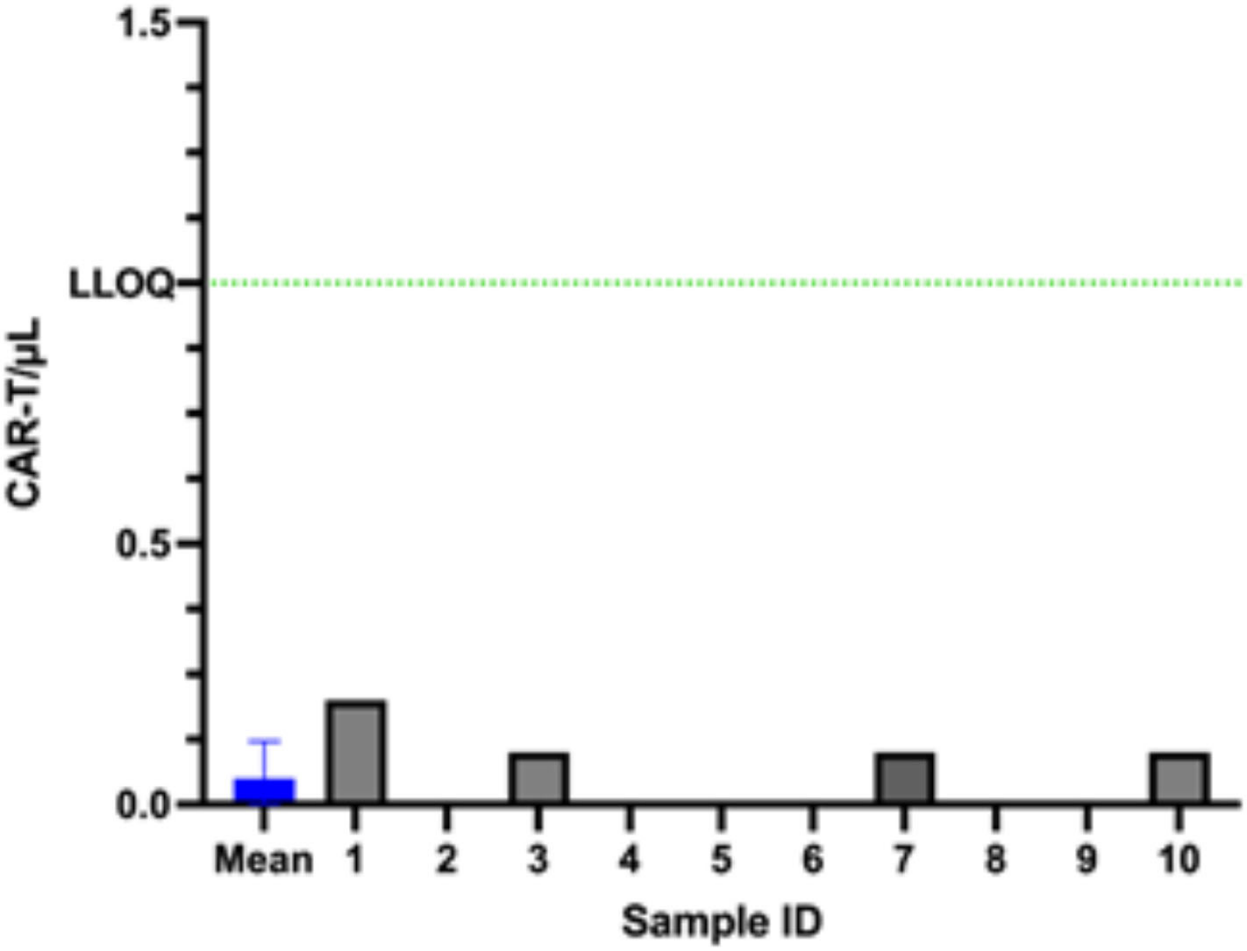
Determination of LOD and LLOQ for the single-step method. CAR-T cell absolute counts were measured in 10 negative control samples from patients not treated with CD19 or BCMA CAR-T cells. The blue line represents the mean background signal. LOD was defined as mean + 3 SD and LLOQ (green dashed line) as mean + 10 SD.

An important question as we moved to a single-step technique with directly coupled reagents and no washing step was whether we would lose sensitivity. As for our first method, we performed CAR-T cells measurements, for both CD19 and BCMA CAR Detection Reagent in blood from 10 different patients that did not receive the corresponding BCMA or CD19 CAR-T cells. Surprisingly we found a lower LOD and LLOQ, with 0.3 and 1.0 CAR-T cells per µL respectively (**Figure 6**).

### Direct comparison of the 2 methodologies

**Figure 7:**
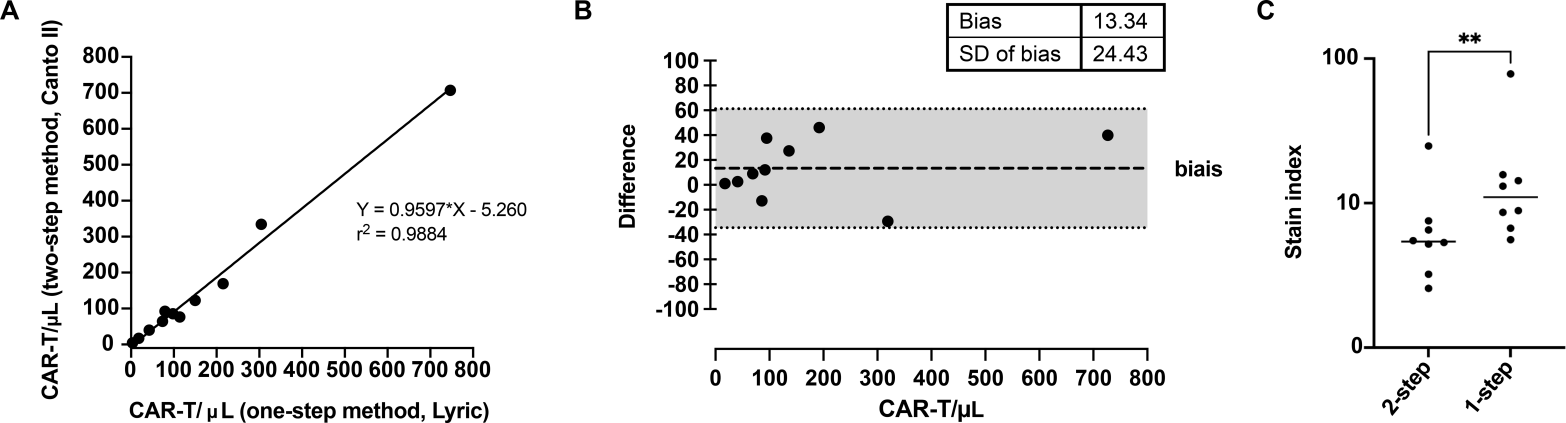
Direct comparison between the two-step and single-step methodologies. **(A)** Linear regression analysis of paired samples (n = 10) showing correlation between absolute CAR-T cell counts obtained with both methods. The coefficient of determination (r²) was 0.9884. **(B)** Bland–Altman plot showing the mean difference between methods and limits of agreement defined as mean ± 2 standard deviations. All values fell within predefined acceptable limits. **(C)** Stain index for eight independent samples stained with both methods. Stain index = ((MFI CAR+ population) – (MFI CAR− population))/(2 × standard deviation of the CAR− population). Bars indicate the mean of each group of samples. ** p ≤ 0.01.

To complete the validation of our updated methodology, we compared results from the two approaches using ten paired samples. The correlation between the two techniques was excellent, with an r² of 0.9884, and this concordance remained robust even at low or very low CAR-T cell concentrations (**Figure 7A**). In the Bland Altman analysis, all measurements fell within the predefined acceptance limits of plus or minus two standard deviations (**Figure 7B**). Furthermore, the single-step technique allowed to a better separation between the positive and negative populations, with a higher staining index (18.9) than the original two-step method (7.6, p=0.008) (**Figure 7C**).

These findings confirm that the single-step protocol, which omits the washing step after CDR staining, performs as reliably as the original two-step method.

## Discussion

We report on the validation of anti-CD19 and anti-BCMA viable CAR-T cell concentration measurement method using flow cytometry techniques that allows to monitor expansion in peripheral blood. Flow cytometry has demonstrated greater convenience for clinical lab routines compared to molecular biology tools like RT-qPCR. However, RT-qPCR remains valuable due to its superior sensitivity, which enables extended monitoring and its feasibility even in lymphopenic conditions seen in CAR-T treated patients or in paucicellular matrices such as cerebrospinal fluid (34). Integrating flow cytometry and molecular quantification approaches would allow more comprehensive pharmacokinetic characterization of CAR-T in these conditions.

We implemented a fast (<1h) and simple method, using only 50µL of sample. The absolute quantification was performed using a single-platform which is more robust, especially when working with thawed products, for which low viability may impact results with dual-platform methods (13). Intra and Inter-assay precisions agreed with published specifications (18, 30) and the results from other groups (35), both for fresh blood samples and for thawed CAR-T products; during validation, we measured a broad range of concentrations in the blood, as well as in thawed CAR-T products. As CAR-T cells are autologous product, i.e different for each patient, batch to batch comparison are hard to consider. Hence, the range is consistent with previously reported data (4, 9) and regularly observed in our daily clinical practice. In the absence of an EQA program, and in order to compare our methodology with a previously published method routinely used at another center (19), we performed an inter-laboratory comparison. However, only CAR-T cell frequencies were analyzed. Absolute CAR-T cell counts were not compared because participating centers used heterogeneous quantification strategies, including single-platform bead-based flow cytometry and dual-platform approaches combining cytometric frequencies with automated hematology analyzer leukocyte counts. These methodologies are not analytically equivalent and may introduce systematic variability, thereby preventing direct comparison of absolute counts. These methodological considerations highlight the importance of harmonizing absolute counting strategies when comparing CAR-T monitoring results across centers.

To further improve robustness and simplification, we implemented a single-step method using directly fluorochrome coupled CAR detection reagent for both BCMA and CD19 CAR-T cells. The single-step (no-wash) method significantly reduces operator workload and errors without compromising precision. Lee W-H et al. developed a similar BCMA CAR-T monitoring method but reported no-wash artefacts, likely reagent-specific (36). Up to date, we did not encounter this issue in our setup. Peinelt et al., observed an imprecision ranging from 0.0-16.8% compared to 0.7-7.9% with our first method (28). Using internal negative controls, we easily identified CAR-positive T cells and validated a 5 CAR-T/µL LLOQ with the two-step method and an improved 1 CAR-T/µL LLOQ with the single-step method, that is in line with LLOQ measured for other single-platform methods developed or adapted in our laboratory (14, 15). Consequently, we have determined that our gating strategy and the flow cytometry technique can detect minimal numbers of circulating CAR-T cells, whether assessed at the very beginning expansion phase following axicel infusion or for very low concentration at long term CAR-T persistence.

With the improved technique, we also sought to refine CAR-T cell characterization, particularly during the early expansion phase. CAR-T cells are not a homogeneous population and can be broadly classified into CD4 or CD8 lineages. CD8 T cells have cytotoxic functions and are essential for antibacterial and antitumor immunity. Several studies suggest that CD8 CAR-T cells play a prominent role immediately after infusion. Talleur et al. reported preferential expansion of CD8 CAR-T cells following CD19 CAR-T infusion in patients with B acute lymphoblastic leukemia, and Galli et al. showed that the CD4 to CD8 ratio in the infused product was a prognostic factor in diffuse large B-cell lymphoma (37, 38). CD4 T cells, also known as helper T cells, have distinct and complementary roles. In a murine model, Bove et al. demonstrated that CD4 CAR-T cells contributed significantly to CRS and were also involved in long-term therapeutic responses (39). In addition, recently commercialized products potential that are alternative to axicel in r/r DLBCL, such as lisocabtagene maraleucel (lisocel), include a fixed CD4 to CD8 ratio of 1:1 in the medicinal product (40). Together, these findings indicate that CD4 and CD8 CAR-T cells have different functions and expansion dynamics. Nevertheless, our analyses remain constrained by its relatively low dimensionality, preventing a more detailed examination of specific cell subsets including maturation state or exhaustion status.

In our study we also validate the clinical significance of CAR-T cells biological monitoring, as circulating CAR-T levels correlate with the objective response rate in a cohort of 29 patients treated with axicel. Consistent with previous reports in DLBCL. In the literature, higher peak blood levels of CAR-T cells have been associated with improved response rates (10, 11), as well as an increased incident of grade ≥ 3 ICANS (4), findings that we partially confirmed in our cohort. The limited sample size, reflecting the early phase of CAR-T cell implementation at our institution, restricted our ability to identify associations beyond objective response. Although several clinical studies have incorporated biological monitoring and reported correlations with outcomes, these data have not yet translated into clear decision-making algorithms for clinicians (3, 35). Mathematical modeling, which integrates tumor load and CAR-T cell expansion data, suggests a model that links early expansion proportionate to tumor load, rather than the persistence of functional circulating CAR-T cells, with a lasting response (41). CAR-T Cells expansion and persistence were recently linked to biphasic neutropenia (42). Further investigation is warranted concerning the recently reviewed Immune effector cell–associated hematotoxicity (ICAHT) (43).

The identification of early biomarkers serves as a valuable tool for clinicians, enabling prompt treatment for patients at a high risk of relapse with immunomodulatory drugs like lenalidomide (29). Therefore, this places impetus to advance the development of innovative techniques with the view to explore additional pharmacometric parameters that can reliably predict CAR-T cells efficacy and toxicity. Other immune parameters both cellular and soluble molecules seem to be influent on the patient outcome, starting from the lymphapheresis composition, as well as the systemic inflammation (evidenced by LDH, ferritin, cytokines and chemokines levels) impairing CAR-T cells expansion, differentiation and function (10, 44, 45). The response to CAR-T cells is dependent on a combination of plural factors, which are intrinsically linked, whose integrative analysis should lead to the establishment of more complex predictive signature. As shown with our cohort, CAR-T immune monitoring in patients’ blood is certainly one major key of the response.

## Conclusion

We have implemented a rapid, standardized, dependable and clinically usable flow cytometry-based approach to repeatedly monitor CD19- and BCMA-targeted CAR-T cells concentrations, enabling real-time tracking of their levels both in circulation and in the thawed product. The first (two-step) method was accredited by the French accreditation committee (COFRAC) in September 2021, recognizing compliance with ISO 15189 international standards. So was the single-step methodology in 2024 that leads to robust results and will be used for follow-up studies. Ongoing efforts to achieve national or international harmonization will enhance data interpretation and facilitate comparisons across various institutions.

## Data Availability

The datasets presented in this article are not readily available because patients data were used to validate our methods and assessed biological findings to clinical responses/toxicities, theses data are not intented for other diffusion than this publication. Requests to access the datasets should be directed to lemariec@ipc.unicancer.fr.

## Glossary

7AAD: 7-Aminoactinomycin D
BCMA: B Cell Maturation Antigen
CAR: Chimeric Antigen Receptor
Cmax: Maximum Concentration
COFRAC: French Accreditation Committee
CRS: Cytokine Release Syndrome
CV: Coefficient of Variation
DLBCL: Diffuse Large B-Cell Lymphoma
EDTA: EthyleneDiamineTetraAcetic Acid
FDG: FluoroDeoxyGlucose
GSPC: Clinical Project Selection Groupe
ICH: International Conference on Harmonization
ISO: International Organization for Standardization
ICANS: Immune effector Cell-Associated Neurotoxicity Syndrome
ICAHT: Immune effector Cell–associated HematoToxicity
LOD: Limit of Detection
LLOQ: Lower Limit of Quantification
PCR: Polymerase Chain Reaction
PET: Positron Emission Tomography
RT: Room Temperature
SD: Standard Deviation
WBC: White blood cells
